# Assessing the preservation of biogenic strontium isotope ratios (^87^Sr/^86^Sr) in the pars petrosa ossis temporalis of unburnt human skeletal remains: A case study from Saba

**DOI:** 10.1002/rcm.9277

**Published:** 2022-03-21

**Authors:** Lisette M. Kootker, Jason E. Laffoon

**Affiliations:** ^1^ Geology & Geochemistry Cluster Vrije Universiteit Amsterdam Amsterdam the Netherlands; ^2^ CLUE+ Vrije Universiteit Amsterdam Amsterdam the Netherlands; ^3^ Faculty of Archaeology Leiden University Leiden the Netherlands

## Abstract

**Rationale:**

Strontium isotope (^87^Sr/^86^Sr) analysis of skeletal remains has become a powerful tool in archaeological studies of human migration and mobility. Owing to its resistance to post‐mortem alteration, dental enamel is the preferred sampling material used for ^87^Sr/^86^Sr analysis in bioarchaeological provenance research, although recent studies have demonstrated that cremated bone is also generally resistant to diagenesis. This paper presents the results of a pilot study exploring the potential of unburnt petrous bone (pars petrosa) as a reservoir of biogenic (diagenetically unaltered) strontium, as the otic capsule or bony labyrinth within the petrous bone is extremely dense and is thought to be unable to remodel after early childhood, potentially providing an alternative for dental enamel.

**Methods:**

From an individual from a colonial‐era (18th century) site on the island of Saba in the Caribbean for whom previous enamel ^87^Sr/^86^Sr results had indicated non‐local origins, multiple locations (*n* = 4) on the petrous were sampled and measured for strontium isotope composition. Saba (13 km^2^) has been extensively mapped for baseline strontium isotopes (*n* = 50) with ^87^Sr/^86^Sr varying from *ca* 0.7065 to 0.7090, whereas enamel ^87^Sr/^86^Sr (*n* = 3) ranged from 0.7104 to 0.7112.

**Results:**

All four petrous ^87^Sr/^86^Sr ratios (0.7111–0.7122) are consistently and considerably higher than the local bioavailable range, and very similar to the enamel ^87^Sr/^86^Sr. These results provide initial evidence that unburnt petrous bones may preserve biogenic strontium, at least in this specific burial context.

**Conclusions:**

While more research in diverse burial conditions is needed to validate this observation, if confirmed, it would have broader implications for sample selection strategies in bioarchaeological studies using the strontium isotope method.

## 1 INTRODUCTION

The human pars petrosa ossis temporalis has become the ‘Holy Grail’ in ancient DNA studies since pioneering research by Pinhasi et al in 2015 showed that relatively elevated quantities of high‐quality endogenous aDNA were preserved in the bony otic capsule of the inner ear.^1^ Recently, the otic capsule, or bony labyrinth, has proven to be valuable in archaeological isotope studies as well.^2^ Due to their increased crystallinity, cremated or calcined archaeological skeletal remains have proven to exhibit the diagenetically unaltered, i.e. original or biogenic, strontium isotope composition (^87^Sr/^86^Sr) at the time of death.^3^, ^4^, ^5^ Due to the extremely slow remodelling rate of the otic capsule compared to the surrounding bone of the petrous or other bones in the skeleton,^6^, ^7^, ^8^ the bony labyrinth of cremated petrous portions (PP) is nowadays considered the equivalent of dental enamel and thus a reliable proxy for the geochemical environment of infancy. As such, calcined otic capsules can be effectively used to provide insights into the childhood (geographical) provenance of cremated burials for which dental enamel is often not well preserved or recovered.^8^, ^9^, ^10^

However, to date, little research has been conducted on the applicability of the pars petrosa or otic capsule in unburnt archaeological skeletal assemblages for provenance studies in cases where dental elements are absent. A major problem in isotopic studies on the apatite component of bone, however, is the process of diagenesis.^11^ The post‐mortem chemical and physical changes that happen to a bone during burial have a negative and irreversible impact on the biogenic strontium isotope composition of bone bioapatite. In contrast to the porous structure of bone, the otic capsule is the densest bone in the human skeleton^12^ and therefore considered to be less prone to diagenetic alterations.

To investigate the effect of diagenesis on the biogenic ^87^Sr/^86^Sr of the otic capsule of unburnt pars petrosae, and its applicability for provenance studies, a unique case study was investigated. In this study the ^87^Sr/^86^Sr^13^ and Sr concentrations of enamel and dentine samples from three molars and of one pars petrosa are presented from an inhumed female from a historic period site on the island of Saba, a special municipality of the Netherlands in the northeast Caribbean. Based on the archaeological, osteoarchaeological and isotopic evidence (elevated, non‐local ^87^Sr/^86^Sr), this individual was interpreted as an individual of African origin, who was enslaved and forced to migrate to Saba in the late 18th century.^13^ The strontium isotope difference between the region of childhood origin (Africa) and the location of death (Saba) is immense. The bioavailable strontium isotope range on Saba (mean: 0.7083; range: 0.7065–0.7090) is much lower than that in much of West and West‐Central Africa, which is underlain by diverse geological formations including large areas of continental bedrock (including cratonic formations) with highly variable but generally higher ^87^Sr/^86^Sr (greater than *ca* 0.710).^14^ Consequently, a diagenetic shift in the otic capsule's ^87^Sr/^86^Sr towards the Saba range should be apparent if the otic capsule is affected by diagenesis. This paper presents the results of this pioneering study and provides directions for future research on the use of unburnt pars petrosae in archaeological mobility studies.

## 2 DIAGENESIS IN PALAEOMOBILITY STUDIES

The effects of diagenesis on the isotopic integrity of archaeological bone and dentine have been the subject of research for the past few decades. Nearly four decades ago, when strontium isotope analysis was first introduced to the field of human biomolecular archaeology,^15^, ^16^ the complicating effects of diagenesis were already acknowledged.^17^, ^18^ In the 1980s, decontamination of (bone) tissue was thought to be accomplished through ‘mechanical excoriation, tissue separation, and phase separation by chemical reaction’ with acetic acid.^16^, ^19^, ^20^ Nevertheless, convincing evidence of diagenetic alteration of bone chemistry and strontium isotope composition was provided shortly after in 1986 by Nelson and colleagues and confirmed by many studies thereafter.^21^, ^22^, ^23^, ^24^

Many early studies of human palaeomobility based on strontium isotope analysis focused on sampling of enamel and bone.^16^, ^25^, ^26^, ^27^, ^28^ Over time, more evidence accumulated demonstrating the higher susceptibility of bone and dentine to diagenesis (relative to enamel) generally, and post‐mortem alteration of biogenic strontium isotope signals more specifically.^21^, ^22^, ^29^ Owing to this, bone has become slowly abandoned as a suitable sampling material in most strontium isotope studies of palaeomobility over the last two decades. Nevertheless, some studies continued to analyse bone and dentine strontium isotope signals as proxies of the local range of bioavailable ^87^Sr/^86^Sr under the debatable assumption that these sampling materials should be reflective of local diagenetic ^87^Sr/^86^Sr^30^, ^31^ or that all diagenetic contaminants were successfully removed.^32^

Until several years ago, dental enamel was considered the only suitable sampling material for archaeological palaeomobility and provenance studies owing to the fact that it does not undergo remodelling once fully mineralized and that is it generally resistant to post‐mortem isotopic alteration. A study by Jørkov and colleagues,^2^ however, highlighted the point that the otic capsule of the petrous portion of the temporal bone is also a suitable proxy for stable isotope studies of the early dietary patterns similar to enamel, as it does not appear to undergo remodelling once fully formed in infancy/early childhood. After pioneering experimental research by Harbeck et al,^3^ another experimental study by Snoeck et al^5^ convincingly demonstrated that the process of cremation at high temperatures (calcination) does not alter biogenic ^87^Sr/^86^Sr, and opened up the strontium isotope method to be applied to cremated remains in archaeological contexts.^9^, ^10^, ^33^, ^34^, ^35^

In fact, the first application of strontium isotope analyses to PP by Harvig et al obtained ^87^Sr/^86^Sr in both cremated and inhumed skeletal remains to assess the reliability of the petrous as a sampling element for strontium isotope mobility studies.^4^ To our knowledge, this study represents the only study conducted to date to measure ^87^Sr/^86^Sr in unburnt PP.^4^ Owing to the high degree of correlation between (premolar) enamel and petrous ^87^Sr/^86^Sr signatures obtained from the same individuals, for both cremated and inhumed remains, the authors concluded that biogenic strontium is retained in archaeological petrous samples and is indicative of childhood origins.^4^ However, although their study apparently included two individuals of non‐local origins, most of the sampled individuals were of local origins. Additionally, premolar enamel crowns develop and mineralize from roughly 2 to 6 years of age after the remodelling of the otic capsule is believed to cease, and thus it is difficult to assess if differences in ^87^Sr/^86^Sr between petrous bone and premolar enamel samples reflect age‐related changes or diagenesis of the former. Furthermore, their study reported the range of bioavailable ^87^Sr/^86^Sr at the national level (Denmark), whereas the local (site) bioavailable ^87^Sr/^86^Sr ranges were not reported. These issues are interpretively problematic as strontium isotope ratios obtained from non‐cremated bones may correlate with those obtained from dental enamel for local individuals for various reasons: e.g. bone diagenesis, absence of migration, migration between isotopically similar locales, etc. For example, similar enamel and bone (PP) ^87^Sr/^86^Sr ratios obtained from the same individual could simply result from the lack of movement (local origins) and/or diagenetic alteration of bone ^87^Sr/^86^Sr.

In this regard, the present study represents a reassessment of the reliability of strontium isotope ratios obtained from inhumed (unburnt/non‐cremated) pars petrosa samples for human palaeomobility research. The research focuses on a single individual recovered from an archaeological context where the local and regional bioavailable strontium isotope ranges are well characterized (*n* = 50 baseline samples) and whose dental enamel ^87^Sr/^86^Sr deviates substantially from the local range indicating distant (non‐local) childhood origins. Combining strontium isotope and strontium concentration analyses of multiple tissues formed at different periods of this individual's life allows us to more robustly assess intra‐individual differential diagenesis and to test the reliability of non‐cremated archaeological PP as a proxy for childhood geographic origins.

## 3 CASE STUDY

**FIGURE 1:**
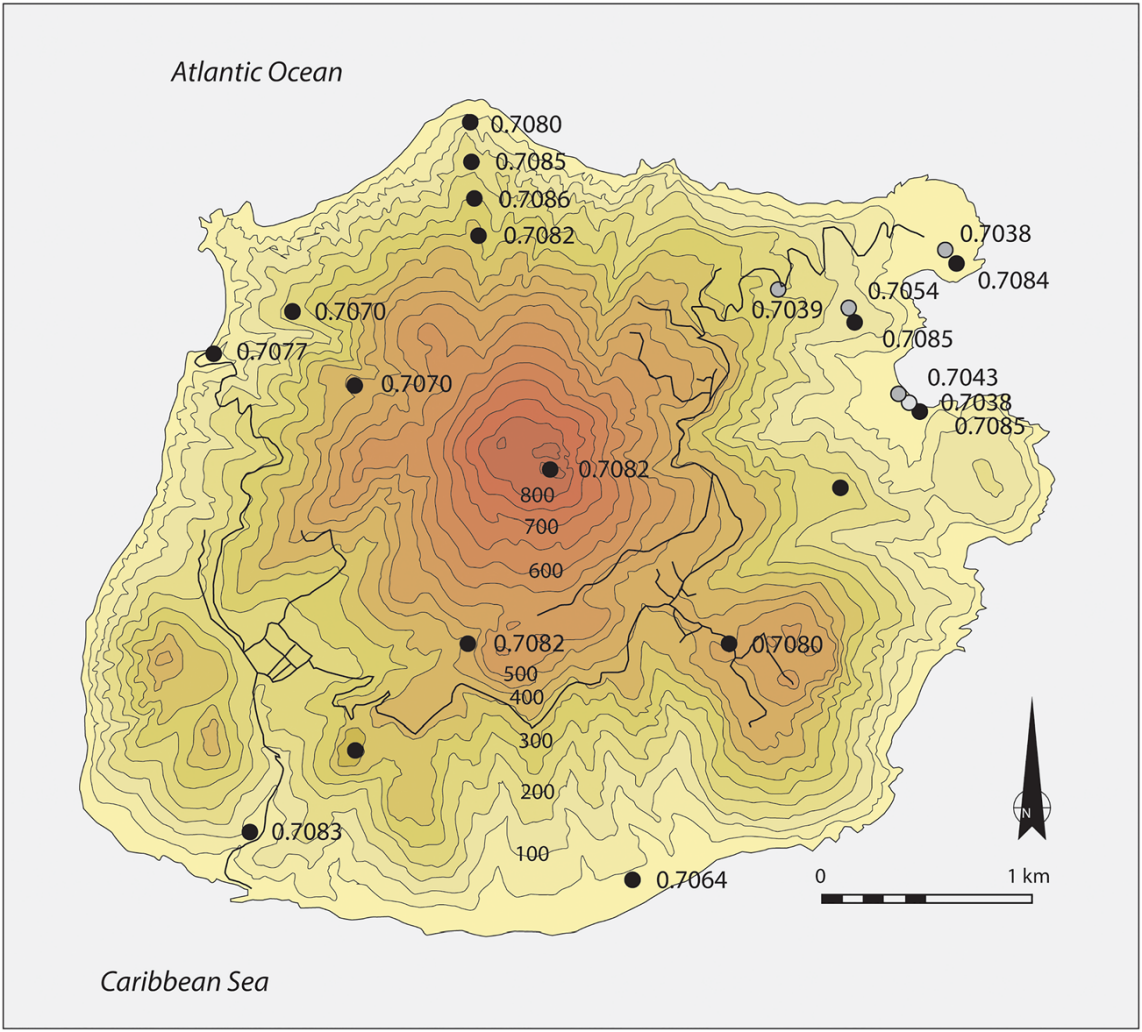
Map of Saba with bioavailable ^87^Sr/^86^Sr^36^, ^41^ [Color figure can be viewed at wileyonlinelibrary.com]

During archaeological excavations in 2015 at the Fort Bay Ridge site on the island of Saba in the northeast Caribbean, performed by the project Nexus1492, Leiden University in collaboration with the Saba Archaeological Centre (SABARC), the remains of an adult female (individual 1) and a foetus (individual 2), dating to the late 18th century AD (cal 1730–1807, 52.3%: IntCal 20, Oxcal v4.4.4, laboratory ID GrA‐68585) were recovered. Strontium isotope analysis revealed that the female was of non‐local origin and may have spent her childhood, at least until the age of *ca* 16–18 years old, in sub‐Saharan Africa.^13^ She was probably a first‐generation enslaved African forcibly migrated to the Caribbean, likely as victim of the transatlantic slave trade. The strontium isotope composition of the geological region she spent her childhood in differs significantly from that of Saba. The three enamel samples (M1, M2, M3) exhibit high strontium isotope ratios, ranging between 0.7105 and 0.7112.^13^ These ratios are extremely elevated compared to the bioavailable ^87^Sr/^86^Sr for both the island of Saba (*ca* 0.7065–0.7090, Figure 1, ^36^) and the Antilles more broadly (*ca* 0.7055–0.7093^37^, ^38^, ^39^). In the Caribbean, including Saba, bioavailable ^87^Sr/^86^Sr ranges were obtained by sampling a diverse array of proxies (archaeological rodent enamel and land snail shells, modern plants and water), and these generally possess low ^87^Sr/^86^Sr ratios (<0.7093) relative to the global range, reflecting variable contributions of Sr from bedrock geology and precipitation/sea‐spray.^40^ The particularly large difference between the human and local bioavailable ^87^Sr/^86^Sr (mean difference of 0.0027 is roughly 150–300 times greater than the typical analytical precision of strontium isotope measurements) makes this case ideal for a study of the effects of diagenesis on the isotopic integrity of the petrous part of the temporal bone, and dentine, in archaeological studies in the Caribbean.

## 4 MATERIALS AND METHODS

### 4.1 Materials

A more detailed description of the site and the osteoarchaeological data of the middle adult (36‐ to 45‐year‐old) female and the *ca* 34‐ to 40‐week‐old foetus is given in Fricke et al.^13^ For this study, the right PP of the adult female was selected for Sr isotope research; the left specimen was of better quality and therefore saved for future aDNA research. The PP was cut through its central axis to create a midmodiolar section through the cochlea using a Buehler (Esslingen, Germany) IsoMet 1000 precision saw. Following the optimized sampling strategy of Veselka et al,^8^ two samples of the cochlea and one of the semicircular canal were taken. In addition, a reference bone sample was collected from an area that is not part of the otic capsule and as such was subject to remodelling during life. The quality of the PP of the foetus was poor and did not allow the PP to be cut midmodiolarly. Instead, the outer surface of the PP was mechanically cleaned, and a random bone sample was taken. In addition to the PP bone samples and the previously published enamel samples from the individual,^13^ three dentine samples were taken from the same three teeth (36, 47 and 28). The tooth roots were mechanically cleaned prior to removal of the dentine samples from the exterior surface of the middle section of the root.

All bone and dentine samples were taken at the Archaeological and Forensic sample preparation laboratory at the Vrije Universiteit Amsterdam using a Proxxon drill equipped with a ball‐shaped diamond‐coated grinding bit and collected in clean glass vials. After each sample, the drill bit was cleaned in Milli‐Q water, 10% HCl and ethanol to prevent inter‐sample contamination. The samples were collected in 6–7 M HCl‐cleaned 2.0 mL Eppendorf^®^ tubes and transferred to the US Federal Standard Class 100 (ISO5) clean laboratory facility at the Vrije Universiteit Amsterdam for Sr purification.

### 4.2 Isotope analysis

Strontium column extraction and sample loading were performed following previously published protocols.^42^ The isotope compositions were measured using a Thermo Scientific™ Triton Plus™ instrument housed at the Vrije Universiteit Amsterdam, the Netherlands. The strontium ratios were determined using a static routine and were corrected for mass fractionation to ^86^Sr/^88^Sr of 0.1194.^43^ The NIST^®^ SRM^®^ 987 Strontium Carbonate reference material gave an average of 0.710258 ± 0.000008 (2*s*) during the course of this study (*n* = 77). The intermediate precision over the period 2017–2021 using this method was 0.710254 ± 0.000018 (2*s*; *n* = 433). All measurements were normalized to an accepted value of 0.710240 for SRM 987. The total procedural blanks (*n* = 5) contained between 28 and 52 pg of strontium. The ^87^Sr/^86^Sr values are reported plus or minus two standard error (2SE), representing the typical measurement precision obtained from 240 cycles of 8.1 s integration time (12 blocks of 20 cycles) within each run.

### 4.3 Concentration measurements

Between 1.3 and 4.1 mg of dentine and enamel powder was transferred into acid‐precleaned inductively coupled plasma tubes, and diluted with 10% HNO_3_ (dilution factor 4500–5500). The acidified samples were measured using a Thermo X‐Series II inductively coupled plasma mass spectrometer at the Vrije Universiteit Amsterdam, the Netherlands. The aliquot solutions were introduced via a quartz dual cyclonic spray chamber equipped with a PFA‐ST MicroFlow nebulizer (Elemental Scientific) with a sample uptake rate of about 100 μL min^−1^. Changes in sensitivity over time were monitored and corrected for using the geological reference material BHVO‐2, which was measured after every second sample (modified after Eggins et al^44^). Strontium concentrations were calculated using a two‐point calibration of blank and BHVO‐2. The intermediate precision determined with multiple geological reference materials was about 10% (2*s*).

## 5 RESULTS

**FIGURE 2:**
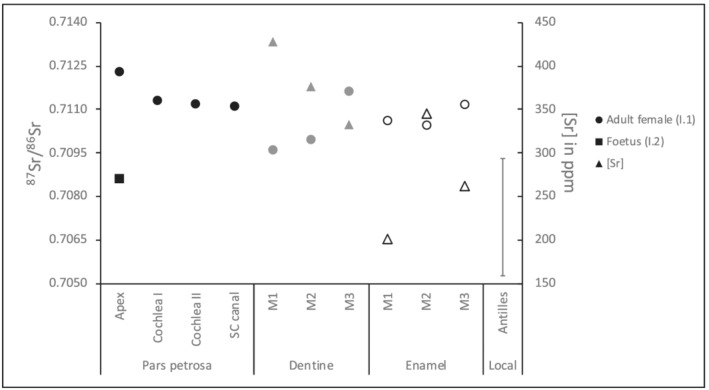
Strontium isotope ratios (^87^Sr/^86^Sr) of enamel, dentine and petrous samples, and strontium concentrations [Sr] of enamel and dentine samples from individuals 1 and 2 from Saba. Key: SC canal, semicircular canal; M1, first molar; M2, second molar; M3, third molar

The results of the strontium isotope analysis and concentration measurements are provided in Table 1 and Figure 2. The three samples collected from the petrous part's otic capsule in the female (individual 1) all provide similar ^87^Sr/^86^Sr, ranging between 0.7111 and 0.7113. The sample taken from a randomly chosen location within the petrous part exhibits a more radiogenic strontium isotope composition: 0.7123. None of these data are compatible with the bioavailable strontium isotope range of Saba.^36^, ^41^ On the contrary, the ^87^Sr/^86^Sr of the randomly selected petrous sample of the foetus (0.7086) matches well with the local ^87^Sr/^86^Sr (median: 0.7084). The strontium isotope compositions of the dentine samples are similar to the previously published dental enamel data (0.7105–0.7112^13^), i.e. much more radiogenic than the Saba signature, ranging from 0.7096 in the M1 (36) to 0.7116 in the M3 (38). The dental strontium concentration data fluctuate between 201 and 345 ppm in enamel and 332 and 427 ppm in dentine, with the dentine Sr concentration generally being higher.

**TABLE 1:** Strontium isotope ratios (^87^Sr/^86^Sr) of enamel, dentine and petrous samples; and strontium concentrations of enamel and dentine samples^a^

ID	Sex	Age at death	Material	Element	Sample	^87^Sr/^86^Sr	2SE	[Sr] (ppm)
Individual 1	Female	36–45 years	Bone	PP	Cochlea	0.711202	0.000007	—
					Cochlea II	0.711320	0.000008	—
					Semicircular canal	0.711118	0.000007	—
					Apex	0.712292	0.000009	—
			Enamel^b^	Molar	36	0.710630	0.000011	201
					47	0.710450	0.000011	345
					28	0.711170	0.000010	262
			Dentine	Molar	36	0.709572	0.000008	427
					47	0.709962	0.000009	375
					28	0.711633	0.000008	332
Individual 2	—	34 weeks *i.u*.	Bone	PP	Random	0.708611	0.000008	—

^a^ PP, petrous part; 2SE, 2 standard error; [Sr], strontium concentration; *i.u*., in utero.

^b^ Data from Fricke et al.^13^

## 6 DISCUSSION

Since the strontium isotope ratios of all three dental enamel samples are significantly higher than the local range, this suggests that the proposed transatlantic migration occurred after childhood (after formation of the third molar enamel which is completed around 11–16 years of age^45^, ^46^). However, the ^87^Sr/^86^Sr of the third molar enamel is somewhat elevated relative to that of the first and second molar enamel samples. In a previous study this was tentatively suggested to indicate a possible prior migration event in adolescence, perhaps within Africa.^13^

The strontium concentrations in the dentine sample increased between 9% in the M2 (27) to 112% in the M1 (26) compared to the enamel samples from the same dental element. Since the Sr concentrations in dentine and enamel are similar during life (see Supporting Information ‐ Data S1), the increase in Sr concentrations is considered to reflect partial incorporation of diagenetic Sr. For the first and second molars, dentine samples possess higher Sr concentrations and lower ^87^Sr/^86^Sr that are intermediate between the corresponding enamel and the median of the local bioavailable ^87^Sr/^86^Sr. This suggests that these dentine samples contain a mix of biogenic and diagenetic strontium. A slightly different pattern is observed for the third molar dentine sample, which has a Sr concentration that is slightly higher than that of the corresponding enamel sample (27%), but exhibits an ^87^Sr/^86^Sr that is actually more radiogenic than the third molar enamel ^87^Sr/^86^Sr. If a diagenetic shift towards the local signature is assumed, the biogenic third molar dentine ^87^Sr/^86^Sr must have been even more radiogenic than 0.7116. This pattern suggests that the third molar dentine is not highly impacted by diagenesis, and lends further support to the notion that this individual may have undergone a previous migration that occurred subsequent to the mineralization of the third molar enamel and during the formation of the third molar root dentine. As third molar roots generally start to develop during late childhood and finish forming at approximately 20 years of age, this may indicate that this purported earlier migration event occurred as an adolescent or young adult. As both the M3 enamel and dentine ^87^Sr/^86^Sr are highly elevated relative to the bioavailable range for the Antilles, this also suggests that this earlier (forced) migration within the African continent occurred prior to the later forced migration to the Caribbean.

**FIGURE 3:**
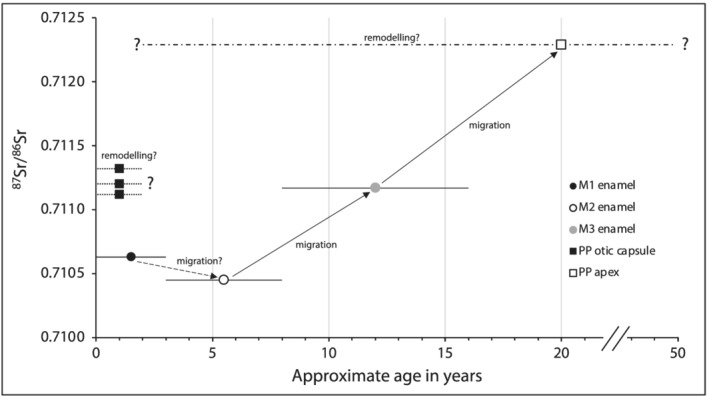
Strontium isotope ratios (^87^Sr/^86^Sr) of enamel and petrous samples plotted relative to approximate age of formation/mineralization. The three otic capsule samples exhibit ^87^Sr/^86^Sr that are more consistent with the third molar (M3) than the first or second molars (M1 or M2), possibly indicating that they reflect a later time period rather than infancy. The single random sample of the petrous has a relatively elevated ^87^Sr/^86^Sr and owing to multiple confounding factors the period that is reflected by this sample is unknown

The ^87^Sr/^86^Sr of the three samples collected from the adult female's otic capsule are substantially higher than the bioavailable strontium signature at the location of burial, and broadly comparable to the ^87^Sr/^86^Sr of the enamel samples. In fact, the strontium isotope composition of the three otic capsule samples is nearly identical to that of the third molar enamel (38) and not to that of the first molar enamel (26) as was expected based on the assumed age at which the PP is thought to stop remodelling (Figure 3). A possible explanation can be found in the autobiography of Individual 1. Fricke et al concluded that she may have suffered from leprosy or another infectious disease.^13^ Some infectious diseases can cause hearing impairment. For example, leprosy or certain viral infections can affect the cochlear system, possibly resulting in (abnormal) bone remodelling in the otic capsule.^47^ Consequently, the otic capsule's ^87^Sr/^86^Sr may not be representative of the first few years of life as more recent ^87^Sr/^86^Sr will have been incorporated during this process, possibly resulting in higher ^87^Sr/^86^Sr more similar to that of the third molar's enamel. Notably, the petrous bone sample that was taken from the apex of the PP exhibits an even higher ^87^Sr/^86^Sr (0.71229), indicating an isotopic shift in the opposite direction of diagenesis. If we assume that this petrous sample (i.e. not from the otic capsule) of the adult undergoes remodelling throughout life, this indicates that this individual must have resided in a region characterized by more radiogenic strontium isotope ratios than the location where she resided in childhood that is recorded in her first and second molar enamel. If so, this lends further support to the two‐migration scenario.

Lastly, the foetal petrous sample's ^87^Sr/^86^Sr falls within the local range of bioavailable ^87^Sr/^86^Sr for the island of Saba. This result could reflect a purely biogenic strontium isotope signal, assuming that the foetal tissue reflects the diet of the mother who resided on Saba throughout her pregnancy. Alternatively, given the rather delicate nature and poor physical condition of the foetal petrous specimen, it may have been particularly susceptible to diagenesis. In the absence of other evidence, it is not possible to determine if the ^87^Sr/^86^Sr of the foetal petrous sample reflects biogenic strontium uptake, post‐mortem diagenesis or some combination of these two processes.

## 7 CONCLUSIONS

The results of this study highlight several important issues with broader relevance for sampling and research design strategies concerning isotope studies of palaeomobility. The pronounced variation in dentine ^87^Sr/^86^Sr recorded within different dental elements of a single individual suggests that dentine is not a suitable sampling material for assessing the local range of bioavailable ^87^Sr/^86^Sr. Although more time‐consuming, expensive and labour‐intensive, sampling of local biosphere samples or archaeological rodents for strontium isotope analyses provides a more reliable and independent assessment of bioavailable ^87^Sr/^86^Sr variation. As long advocated by several research groups, analysis of Sr concentrations (in conjunction with Sr isotope analysis) can provide important complementary information for interpreting strontium isotope data obtained from archaeological skeletal remains and is particularly effective for assessing differential diagenesis. The sampling of multiple tissues and different dental elements can provide much more information concerning the age at which migrations occurred and more insight into individual life histories than sampling strategies that focus solely on a single isotope analysis per individual.

The results of this study provide strong evidence that unaltered, biogenic ^87^Sr/^86^Sr is retained in (unburnt) pars petrosa; at least in this particular archaeological burial context. It is, however, important to study the osteological biography of the individuals in detail prior to analysis. Different diseases or conditions, such as leprosy, osteoporosis and otosclerosis, may result in hearing loss of cochlear origin, possibly due to remodelling or demineralisation of the otic capsule.^47^, ^48^, ^49^ Consequently, the otic capsule's ^87^Sr/^86^Sr may not be fully representative of the early years of life.

Perhaps the most unexpected result of this study was that a sample taken from a random portion of the petrous, outside of the otic capsule, not only seems to possess a biogenic ^87^Sr/^86^Sr but that this enriched ^87^Sr/^86^Sr signal is consistent with that of the M3 dentine and tentatively supports the identification of a previous migration occurring after childhood but prior to the forced migration from Africa to the Caribbean. This is consistent with the historical data showing that in the mid‐18th century the transatlantic slave trade intensified and increasing numbers of individuals were taken captive in the interior of West and West‐Central Africa and later brought to coastal ports for subsequent sale and transshipment across the Atlantic (see Trans‐Atlantic Slave Trade database at slavevoyages.org).

To be clear, we are not advocating that the otic capsule, or the pars petrosa, of inhumed remains are ideal sampling targets for strontium isotope analyses of archaeological skeletal remains. However, the results of this study support the main conclusions of a previous study^4^ indicating that biogenic ^87^Sr/^86^Sr may be retained in inhumed pars petrosa in certain archaeological contexts. Although we expect that dental enamel will remain the preferred sampling material for most isotope studies of human palaeomobility, the pars petrosa may provide an alternative sampling element in cases where teeth are not preserved or are otherwise unavailable for analyses, and can potentially be sampled in conjunction with aDNA studies which increasingly target this skeletal element anyway. Although the results of the present study and those of the previous study from Harvig and colleagues^4^ seem promising, it is necessary to conduct more research into differential diagenesis in a wider range of archaeological burial contexts before strontium isotope analysis of inhumed pars petrosa can be more broadly applied in archaeological palaeomobility studies.

## Supporting information




**Data S1.** Supporting Information

## Data Availability

The archaeological isotope data are available in this paper and Fricke et al^13^ and are shared in the open access and collaborative isotope database IsoArch (isoarch.eu).^50^
