# Supplementary material for: Assessing the preservation of biogenic strontium isotope ratios (87Sr/86Sr) in the pars petrosa ossis temporalis of unburnt human skeletal remains: A case study from Saba
Source: Rapid Commun Mass Spectrom. 2022 Mar 21;36(10):e9277. doi: 10.1002/rcm.9277 (PMC9287042; doi:10.1002/rcm.9277)
Supplement: Supplementary file 1 — Data S1. Supporting Information [file RCM-36-0-s001.docx]

**Supplementary data**

|  |  | **WGS84** | |  |  |  |  | **[Sr] in ppm** | | |
| --- | --- | --- | --- | --- | --- | --- | --- | --- | --- | --- |
| **Province** | **Location** | **Lat.** | **Lon.** | **Sample ID** | **^87^Sr/^86^Sr** | **2SE** | **Reference** | **Enamel** | **Dentine** | **Difference E-D** |
| Noord-Brabant | Breda | 51.59 | 4.78 | Breda 1 | 0.709513 | 6 | Kootker et al., 2020 | 88.2 | 42.5 | -45.7 |
| Noord-Brabant | Wouw | 51.52 | 4.39 | Breda 13 | 0.709605 | 8 | Kootker et al., 2020 | 47.8 | 54.1 | 6.2 |
| Noord-Brabant | Prinsenbeek | 51.60 | 4.71 | Breda 8 | 0.709094 | 7 | Kootker et al., 2020 | 39.2 | 29.3 | -9.9 |
| Zuid-Holland | Rotterdam | 51.92 | 4.49 | Erasmus 12 | - | - | This study | 96.6 | 70.8 | -25.8 |
| Zuid-Holland | Strijen | 51.75 | 4.55 | Erasmus 14 | 0.709288 | 30 | Kootker et al., 2020 | 44.1 | 44.6 | 0.5 |
| Zuid-Holland | Rotterdam | 51.92 | 4.49 | Erasmus 3 | 0.709132 | 13 | Kootker et al., 2020 | 62.5 | 48.2 | -14.3 |
| Zuid-Holland | Rotterdam | 51.92 | 4.49 | Erasmus 7 | - | - | This study | 53.8 | 43.0 | -10.8 |
| Noord-Brabant | Riel | 51.53 | 5.03 | Folter 3 | 0.709572 | 8 | Kootker et al., 2020 | 69.3 | 44.9 | -24.4 |
| Friesland | Leeuwarden | 53.20 | 5.80 | Friesland 10 | 0.708980 | 6 | Kootker et al., 2020 | 20.9 | 43.6 | 22.7 |
| Noord-Brabant | Geffen | 51.74 | 5.46 | Geldermalsen 7 | 0.709409 | 8 | Kootker et al., 2020 | 42.5 | 43.4 | 0.9 |
| Limburg | Klimmen | 50.89 | 5.98 | Heerlen 5 | 0.709323 | 8 | Kootker et al., 2020 | 58.2 | 43.4 | -14.8 |
| Limburg | Venray | 51.53 | 5.97 | Helmond 2 | 0.709711 | 7 | Kootker et al., 2020 | 81.5 | 65.3 | -16.2 |
| Noord-Brabant | Helmond | 51.48 | 5.66 | Helmond 5 | 0.709169 | 6 | Kootker et al., 2020 | 49.2 | 36.3 | -12.8 |
| Noord-Brabant | Helmond | 51.48 | 5.66 | Helmond 7 | 0.709553 | 8 | Kootker et al., 2020 | 27.9 | 65.2 | 37.3 |
| Limburg | Maastricht | 50.85 | 5.69 | Maastricht 16 | 0.709682 | 7 | Kootker et al., 2020 | 39.2 | 38.6 | -0.7 |
| Limburg | Maastricht | 50.85 | 5.69 | Maastricht 6 | 0.709328 | 12 | Kootker et al., 2020 | 66.6 | 50.9 | -15.7 |
| Zuid-Holland | Voorschoten | 52.12 | 4.44 | Nathan | 0.710420 | 7 | This study | 72.7 | 50.4 | -22.3 |
| Friesland | IJlst | 53.03 | 5.66 | Sneek 1 | 0.709058 | 8 | Kootker et al., 2020 | 55.7 | 47.3 | -8.4 |
| Overijssel | Steenwijk | 52.79 | 6.12 | Steenwijk 7 | 0.709086 | 9 | Kootker et al., 2020 | 45.3 | 48.8 | 3.5 |
| Noord-Brabant | Veldhoven | 51.70 | 5.30 | Veldhoven 16 | 0.709461 | 8 | Kootker et al., 2020 | 36.9 | 46.6 | 9.7 |
| Zeeland | Middelburg | 51.50 | 3.61 | Zeeland 8 | 0.709187 | 9 | Kootker et al., 2020 | 76.1 | 46.3 | -29.8 |

Reference

Kootker LM, Plomp E, Ammer STM, Hoogland V, Davies GR. Spatial patterns in ^87^Sr/^86^Sr ratios in modern human dental enamel and tap water from the Netherlands: Implications for forensic provenancing. *Science of The Total Environment.* 2020;729:138992.
